# Ketamine for negative and depressive symptoms in schizophrenia: the evidence so far

**DOI:** 10.3389/fpsyt.2026.1766485

**Published:** 2026-03-16

**Authors:** C. M. Diendorfer, C. Bum, A. Weidenauer, U. Sauerzopf, L. Bartova, I. Dajic, L. Müller, D. Rujescu, N. Praschak-Rieder, M. Willeit

**Affiliations:** 1 Department of Psychiatry and Psychotherapy, Division of General Psychiatry, Medical University of Vienna, Vienna, Austria; 2 Comprehensive Center for Clinical Neurosciences and Mental Health, Medical University of Vienna, Vienna, Austria; 3 Department of Psychiatry and Psychotherapy, Klinik Hietzing, Vienna Health Care Group, Vienna, Austria

**Keywords:** depressive symptoms, glutamate, ketamine, negative symptoms (schizophrenia), schizophrenia

## Abstract

Schizophrenia (SCZ) is a severe psychiatric condition characterized by positive symptoms such as hallucinations or delusions, as well as negative symptoms such as apathy, anhedonia and avolition. Given their chronic nature and limited response to current treatments, managing negative symptoms is a significant challenge for healthcare providers. Furthermore, many individuals with SCZ suffer from depressive symptoms during the course of their illness, which can be difficult to distinguish from negative symptoms as their clinical expression often overlaps. Ketamine, a N-methyl-D-aspartate (NMDA) receptor antagonist, has gained popularity as a rapid and effective treatment for treatment-resistant depression. So far, there have been no randomized controlled trials on the use of ketamine for depressive and negative symptoms in patients with psychotic disorders. However, while some authors have labeled the short-term psychotropic effects of ketamine as “psychotomimetic,” individual case reports have shown promising antidepressant effects without provoking psychotic symptoms in patients with severe psychotic depression and SCZ. Ketamine-induced dissociative phenomena typically subsided spontaneously within one to two hours after administration. This review will primarily focus on potential advantages and risks of ketamine in patients with SCZ with a particular emphasis on its role as a possible treatment for negative and depressive symptoms.

## Background

Schizophrenia (SCZ) is a psychiatric disorder typically manifesting after puberty or in early adulthood. It is associated with considerable suffering, socioeconomic burden, and significant excess mortality, particularly among younger patients (1, 2).

Symptoms of SCZ are categorized into positive, negative and cognitive symptoms (3–5). Positive symptoms, such as delusions, hallucinations and disorganized thinking are most prominent during the acute phase of the illness, also known as psychosis. Negative symptoms, which include blunted affect, reduced speech, diminished motivation, social withdrawal, and anhedonia, are associated with a dysfunction in reward processing and motivated behavior. They contribute substantially to long-term impairment and poor outcomes in patients with SCZ, as they usually do not respond well to conventional antipsychotics. Interestingly, negative symptoms occur early in the disease and can often be detected already in the prodromal stage of SCZ (3, 6). However, in many cases they are not noticed or addressed until symptoms of full-blown psychosis have subsided (7). Furthermore, it is not uncommon for individuals with severe negative symptoms to exhibit these symptoms without overt signs of depressed mood, sadness, pessimistic thoughts or suicidal ideation. Consequently, while depressive and negative symptoms often co-occur, their overlap in terms of observable characteristics is limited.

## The pathogenesis of positive and negative symptoms

Several major neurotransmitter pathways are implicated in the pathophysiology of SCZ. A malfunction of dopaminergic neurotransmission, particularly in the mesolimbic pathways, has become one of the most prominent explanatory models regarding the etiology of psychosis (2). Neuroimaging studies using positron emission tomography (PET) or single-photon emission computer tomography (SPECT) have repeatedly observed a direct relationship between the increase in positive symptoms and the amount of dopamine (DA) released by an *d*-amphetamine (AMPH) or methylphenidate (MPH) challenge (8–10). Results from experiments studying DA synthesis and storage capacity using radiolabeled DA precursor molecules, such as [^18^F]Fluorodopa ([^18^F]FDOPA), form a complementary line of evidence supporting the role of excess subcortical DA signaling in the pathogenesis of psychotic symptoms of SCZ (11). While the role of increased DA functioning in acute psychosis is nowadays generally accepted, recent imaging studies were able to relate negative symptoms of SCZ to low DA functioning by showing an inverse relationship between indices of DA functioning and the expression of negative symptoms (12–14). Using an oral AMPH challenge, and the dopamine D_2/3_-receptor agonist PET radioligand [^11^C]-(+)- 4-propyl-9-hydroxynaphthoxazine ([^11^C]-(+)-PHNO) Weidenauer et al. observed a strong correlation between the Positive and Negative Syndrome Scale (PANSS) (15) negative symptom item “emotional withdrawal” and [^11^C]-(+)-PHNO binding in the putamen of drug-naïve patients with first episode psychosis (14). Since [^11^C]-(+)-PHNO is sensitive towards fluctuations in extracellular DA levels (16–18), these results indicate that emotional withdrawal is more intense when DA levels in the putamen are low. In good agreement with this finding, Eisenberg et al. have observed negative correlations between [^18^F]FDOPA uptake and the expression of negative symptoms in the exact same anatomical region in two independent cohorts of drug-free patients with SCZ (12, 19). Furthermore, a study using an oral MPH challenge in subjects at clinical high risk for SCZ found diminished levels of DA release in the ventral striatum of subjects with pronounced negative symptoms (13). However, beyond the dopaminergic system, increasing evidence implicates additional neurotransmitter systems in the pathogenesis of SCZ, such as glutamate and gamma-aminobutyric acid (GABA) (2).

The N-methyl-D-aspartate (NMDA) receptor hypofunction hypothesis of psychosis suggests that reduced glutamatergic activity at NMDA receptors, particularly on GABAergic interneurons in the prefrontal cortex, may lead to cortical disinhibition and downstream dysregulation of subcortical neurotransmitter systems. This disinhibition may result in excessive excitatory output from glutamatergic pyramidal neurons, which project to midbrain dopamine neurons, thus leading to increased DA release (20–22). Within these fronto-striatal circuits, glutamatergic, GABAergic, and dopaminergic systems seem to interact in complex feedback loops (2, 4, 22).

To examine the role of glutamatergic dysfunction in SCZ more directly, previous studies have investigated, for example, post-mortem brain tissue or cerebrospinal fluid (CSF) of patients with SCZ. Some studies have shown molecular abnormalities in glutamatergic structures in SCZ patients post mortem (23–25). Findings on CSF glutamate levels in patients vs. healthy controls are inconsistent and seem to depend on antipsychotic medication and methods used to determine glutamate concentrations (26–29).

Neuroimaging studies using proton magnetic resonance spectroscopy (¹H-MRS) to detect glutamate (Glu), glutamine (Gln) and their combination (Glx) have provided additional non-invasive insights into *in vivo* glutamatergic abnormalities in SCZ. Across multiple studies, elevated Glu and Glx levels have been observed in subcortical regions such as the basal ganglia (30, 31). Additionally, according to a meta-analysis by Merritt et al., Glx to creatine ratio was positively associated with overall symptom severity, as well as negative symptom severity, especially in the medial temporal lobe (32). However, other meta-analyses have come to the conclusion that there is considerable heterogeneity in ¹H-MRS findings on the glutamate system in SCZ (33, 34). It appears that factors such as illness stage, brain region and antipsychotic treatment may contribute to these heterogenous results. Thus, while ¹H-MRS data somewhat support the notion that glutamatergic dysfunction is involved in the pathogenesis of SCZ, the glutamate findings remain inconclusive and insufficient to delineate a unified pathophysiological model for SCZ (2, 4, 30, 32, 35).

In conclusion, an expanding body of evidence suggests a connection between negative symptoms of SCZ and deficits in subcortical DA signaling. This implies that alterations in DA signaling in SCZ are not solely depending on the presence of acute psychotic symptoms (10). In contrast, dopamine abnormalities are also linked to negative symptoms and appear to involve a simultaneous occurrence of increased and relatively decreased DA function in subcortical dopaminergic brain regions. Given the significant role of frontal cortex glutamate neurons in regulating subcortical DA transmission (12, 14, 36, 37), targeting frontal cortex efferents with glutamatergic drugs emerges as a promising approach for addressing subcortical DA dysfunction.

## Glutamate system based treatments for schizophrenia

Based on the aforementioned NMDA receptor hypofunction model, various pharmacological strategies have been explored over the years to modulate glutamatergic neurotransmission in SCZ. In 2007 Patil et al., investigated LY2140023, a selective metabotropic glutamate 2/3 receptor (mGlu_2/3_) agonist. Compared to placebo, individuals receiving the study medication reported statistically significant reductions in both positive and negative symptoms and LY2140023 was found to be safe and well-tolerated in patients with SCZ (38). In 2013, a multicenter, randomized, open-label phase 2 clinical trial assessed LY2140023 in individuals with moderately severe SCZ, characterized by pronounced negative symptoms and functional impairment. Compared to those receiving standard care (olanzapine, risperidone, or aripiprazole), a significantly higher proportion of patients in the LY2140023 group discontinued the study due to perceived lack of efficacy (39). Additionally, another study from 2011 found that LY2140023 does not demonstrate superior effectiveness compared to placebo or 15mg of olanzapine, as measured by PANSS positive and total scores at 4 weeks (40). Furthermore, a recently published meta-analysis by Aboushawareb et al. confirmed that LY2140023 has no relevant therapeutic effects in the treatment of SCZ (41).

N-acetyl cysteine (NAC), a synthetic compound mainly used as a mucolytic agent in respiratory conditions, also increases extracellular glutamate and was administered to patients with persistent SCZ during a 12-week, double-blind, randomized, placebo-controlled clinical trial evaluating the efficacy of 1200 mg N-acetyl cysteine as a supplementary therapy to standard antipsychotic drugs. The PANSS general and total score for the NAC group decreased over time, while the placebo group worsened clinically (42).

Additionally, some evidence suggests that memantine, a NMDA receptor antagonist and a widely used treatment for Alzheimer’s disease, could be used as an adjunctive therapy alongside conventional antipsychotics to reduce positive and negative symptoms of SCZ (43–47). However, other research has failed to demonstrate significant effects (48, 49). Given the considerable heterogeneity of existing studies and the limited amount of current evidence, larger randomized controlled trials are needed.

Overall, despite evidence suggesting glutamate dysfunction in SCZ, earlier investigations using glutamatergic agents have yielded inconclusive results, indicating the necessity of exploring novel glutamate-modulating treatment approaches to improve therapeutic outcomes.

## Ketamine: mechanism of action

Ketamine, a NMDA receptor antagonist, is an acrylcycloalkylamine and a derivative of phencyclidine (PCP). Depending on the administered dose, it shows dissociative anaesthetic, analgesic or sedative features and therefore has various clinical applications (50). Anesthesia is usually achieved by administering doses between 1–2 mg/kg body weight. In comparison, analgesic effects occur in a dosage ranging from 0,15 – 0,25 mg/kg, while antidepressive effects are observed at doses of 0,2–1 mg/kg (51). Ketamine is a chiral compound and consists of two optical isomers, the S(+)enantiomer as well as the R(-)enantiomer (52). S-ketamine has a higher affinity for the NMDA receptor and therefore greater anaesthetic and analgetic potency compared to the R(-)isomer (52–54). Furthermore, plasma clearance of S-ketamine seems to be greater than that of R-ketamine (55). Ketamine in its free base form is a lipid-soluble molecule which can easily cross the blood-brain barrier, resulting in rapid onset of its effects (50). It can be administered via multiple routes, orally, nasally, and intravenously. However, the oral bio-availability is poor due to extensive first-pass mechanisms (52, 56).

Ketamine modulates glutamate neurotransmission through various mechanisms: it directly inhibits NMDA receptors, with a preference for the GluN2B subunits, either at synaptic sites or extra-synaptically. By that, it selectively inhibits NMDA receptors located on GABAergic interneurons. Additionally, it is further involved in the activation of α-amino-3-hydroxy-5-methyl-4-isoxazole-propionic acid receptors (AMPARs) (54, 55).

As mentioned before, NMDA receptor antagonists may lead to downstream dysregulation of subcortical neurotransmitter systems, such as DA. Studies in humans and non-human primates (57–59) observed an induction in DA release into the extracellular space following ketamine administration (59). Furthermore, ketamine seems to enhance synaptogenesis in the prefrontal cortex, contributing to its apparent antidepressant effects (60). When co-administered with AMPH, it further facilitates DA release (61, 62). Considering its beneficial effects in affective disorders (63), and along the idea that it might help to rebalance the blunted DA transmission associated with negative symptoms, S-ketamine could thus hold therapeutic potential in depressive and negative symptoms of SCZ.

## Psychotropic vs. psychotomimetic effects of ketamine

The term “psychotropic” refers to the ability of a substance to have an effect on mental functions, including mood, perception or behavior. This category encompasses both therapeutic agents such as antidepressants and antipsychotics, and recreational drugs. In contrast, the term “psychotomimetic” describes the capacity of a substance to induce symptoms resembling psychosis, such as hallucinations, delusions or thought disorders.

The concept of “psychotomimetic” effects was vividly discussed in the context of PCP. Synthesized in 1956 for an anesthetic program, PCP was initially a promising new substance because of its lack of respiratory depression according to preclinical studies. The notion that PCP, then known as “Sernyl”, has “psychotomimetic” effects was investigated in studies by Luby et al. and Rosenbaum et al. who compared Sernyl with LSD-25 and amobarbital in terms of their “psychotomimetic” potential. They identified Sernyl as a model substance for SCZ due to its ability to induce reductions in primary attention and motor function, as well as symptoms of depersonalization, subjective disorganization, and hallucinations, similar to those observed with the other substances tested (64, 65). However, Ban et al. later concluded that Sernyl does not display „psychotomimetic” or „schizophrenomimetic” effects in the true sense, but rather amplifies individual psychopathological patterns, although some symptoms exhibited may resemble the positive symptoms of SCZ (66). After its introduction as an anesthetic, PCP gained notoriety as a drug of abuse (“angel dust”) that was linked to violent behavior in multiple reports in the popular press and case descriptions in medical journals. Due to the high propensity of PCP for inducing adverse effects in clinical settings, such as violent emergence reactions after anesthesia, research aimed at the development of a better tolerated alternative with a chemical structure similar to PCP. Ketamine, then known as “Ketalar”, was eventually developed and Sernyl was finally replaced on the market (67).

In the following years, media reports on PCP and various publications (68–70) have perpetuated and reinforced the belief that NMDA antagonists, including ketamine, may mimic or exacerbate endogenous psychosis. In the literature, the glutamate hypothesis of SCZ is often associated with the effects of NMDA receptor antagonists or supported by such effects. The PCP or NMDA antagonist model of SCZ has become a frequently used animal model, as PCP, ketamine, and other NMDA antagonists (e.g., MK-801) induce a phenotype in laboratory animals claimed to resemble psychotic symptoms in humans, including cognitive impairments and working memory deficits (71, 72). However, given that ketamine is a dissociative anesthetic agent, the term “psychotomimetic” used in this context warrants critical reflection on the question whether dissociative phenomena such as illusory perceptual changes represent psychotic symptoms in a true sense. Notably, ketamine’s effects regularly include disturbed consciousness and orientation ataxia, blurred speech, and partial or full amnesia, all of which are characteristic symptoms of organic brain syndrome or delirium, but not SCZ (73).

## Ketamine in therapeutic contexts

In 2000, Berman et al. conducted the first double-blind, placebo-controlled clinical study investigating the potential benefits of ketamine for major depressive disorder (MDD). Their study involved the administration of a single dose of ketamine hydrochloride at 0.5 mg/kg body weight and demonstrated a significant reduction in scores on the Hamilton Depression Rating Scale (HDRS) (74). Furthermore, a study by Price et al. identified rapid and beneficial effects on suicidal ideation (75). In subsequent years, numerous studies were conducted following these initial observations (76–78).

However, the glutamate hypothesis as an explanatory model for psychosis has led to a reluctance to utilize ketamine in patients exhibiting psychotic symptoms, not only in SCZ, but also affective disorders accompanied with psychotic features, likely due to concerns about exacerbating these symptoms. As a result, depressed patients with psychotic symptoms have typically been excluded from ketamine studies.

A review by Veraart et al. concluded that short-term ketamine treatment appears safe and potentially beneficial in patients with psychotic depression or a history of psychosis. Dissociative effects were moderate and self-limiting, and in several cases, comorbid psychotic symptoms improved or even resolved entirely following treatment with ketamine or S-ketamine (79).

In 2016 da Frota Ribeiro et al. successfully treated two patients with psychotic depression with intravenous ketamine. They observed more dissociative but not psychotic symptoms in patients with a history of psychosis after intravenous ketamine, compared to those without a history of psychosis. Dissociative effects were self-limited and lasted for approximately 40 minutes (80).

Furthermore, Pennybaker et al. reported these dissociative phenomena to be transient also in psychotic depression, and that the severity of ketamine’s dissociative effects may diminish with repeated administration (81).

In 2018, Ajub & Lacerda conducted a case series in which four patients with psychotic depression received either intravenous or subcutaneous ketamine (0.5 mg/kg body weight). One of these patients was diagnosed with schizoaffective disorder, depressive type. The patients reported mild dissociative symptoms shortly after ketamine administration, which usually subsided within two hours after administration. None of the patients experienced an exacerbation of psychotic symptoms following treatment. Notably, three of the patients even experienced a marked improvement in psychotic symptoms (82). In another case report in 2022, Carter et al. describe a patient with psychotic depression who received 14 treatments of intranasal S-ketamine over a three-month period. Initially, the patient suffered from anhedonia, sleep disturbances, suicidal ideation, and auditory hallucinations. Following treatment, the affective symptoms improved and even the psychotic features resolved (83). In line with this, a case series by Gałuszko-Wę̨gielnik et al. also demonstrated the safe and effective use of intravenous ketamine (0.5 mg/kg bodyweight) as an add-on treatment in patients with treatment-resistant depression with psychotic features, reporting rapid antidepressant and anti-suicidal effects without exacerbation of psychotic symptoms (84).

A growing body of evidence also shows promising results for ketamine as a treatment of catatonic symptoms. A recently published systematic review by Caliman-Fontes et al. summarized the existing evidence: Ketamine was found to be both well-tolerated and effective, with no reports of exacerbation of psychotic symptoms. Mood disorders represented the most common underlying psychiatric diagnoses among the included cases. Notably, in patients with SCZ ketamine was primarily used as an anesthetic agent during electroconvulsive therapy (ECT), rather than as a stand-alone therapeutic intervention (85). In contrast, a recently published case report described the use of intravenous ketamine (0.5 mg/kg over 40 minutes) as the sole treatment for catatonia in a patient with SCZ, without concurrent ECT. The patient showed a satisfactory clinical response, and no exacerbation of psychotic symptoms was observed (86).

In addition to its growing use in the treatment of catatonia, ketamine is also increasingly being investigated for its therapeutic potential in addressing depressive and negative symptoms in patients with a diagnosis of SCZ. While many earlier studies focused on its use in psychotic depression, recent reports have begun to explore its efficacy within the context of SCZ itself (see **Table 1**).

**Table 1:** Clinical studies using ketamine for psychotic disorders..

Clinical study	Authors	Patients	Comedication	Duration	Dosage	Main results
Rapid Antidepressant Effect of S-Ketamine in Schizophrenia	Bartova L et al., 2018	1 female patient suffering from SCZ with a severe post-psychotic depression	Venlafaxine 300 mg Pregabalin 600 mg Valproate 1000 mg Clozapine 400 mg Risperidone 8 mg	Intravenously administered ketamine over 30 min weekly for 3 weeks in total	0.22 mg/kg body weight (first time) then 0.33 mg/kg body weight (thrice weekly for three weeks)	Good response, reduction in depressive symptoms, no exacerbation of psychotic symptoms
Efficacy of Esketamine in the Treatment of Negative Symptoms in Schizophrenia – A Case Series	Nunes M et al., 2018	6 SCZ patients with a severe impairment related to negative symptomatology	Clozapine up to 700mg Risperidone 4mg Olanzapine 25mg Topiramate 50mg Sertraline 50mg Lithium 900mg	Subcutaneously administered ketamine, once a week, for four weeks	0.5 mg/kg body weight (first week) 0.75 mg/kg body weight (second week) 1 mg/kg body weight (third and fourth week)	Reduction in negative symptoms, moderate decrease in positive symptoms no exacerbation of psychotic symptoms
Adjunct Ketamine Treatment Effects on Treatment-Resistant Depressive Symptoms in Chronic Treatment-Resistant Schizophrenia Patients are Short-Term and Disassociated from Regional Homogeneity Changes in Key Brain Regions – A Pilot Study	Ye J et al., 2019	15 chronic treatment-resistan SCZ with treatment‐resistant depressive symptoms	Not mentioned	Intravenous ketamine 4-week treatment	0.5 mg/kg body weight	Reduction in depressive symptoms, antidepressive effects not maintained after one week, no exacerbation of psychotic symptoms
Adjunct Ketamine Treatment of Depression in Treatment-Resistant Schizophrenia Patients is Unsatisfactory in Pilot and Secondary Follow-Up Studies.	Zhuo,C et al., 2020	15 chronic treatment‐resistant SCZ patients with treatment‐resistant depressive symptoms	Not mentioned	Intravenously administered ketamine 9 times over a period of 25 days	0.5 mg/kg body weight	Antidepressive effects from treatment in pilot-study no longer maintained, no exacerbation of psychotic symptoms

A case report by Bartova et al. describes a young patient with SCZ with post-psychotic depression who received three intravenous administrations of S-ketamine over three weeks, resulting in substantial antidepressant and anti-suicidal effects. The Montgomery-Åsberg Depression Rating Scale (MADRS) scores decreased from 50 to below 10 points, with no significant worsening of positive symptoms, as measured by PANSS–Positive Symptoms Subscale (87). Furthermore, a subsequent pilot study by Ye et al. investigated ketamine’s effects on treatment-resistant depressive symptoms in 15 patients with chronic treatment-resistant SCZ. Participants received 0.5 mg/kg body weight of intravenous ketamine every three days for 4 weeks, resulting in a 64% reduction in Calgary Depression Scale for Schizophrenia (CDSS) scores and a 30% reduction in PANSS general psychopathology subscale scores (baseline score = 29.90). Only one patient reported visual hallucinations during the initial 30 minutes of treatment; other participants did not experience hallucinations (88).

Approximately two months later, Zhuo et al. repeated the intervention with the same 15 patients to assess long-term effects of ketamine. Ketamine was administered intravenously at 0.5 mg/kg body weight over one hour on multiple days following the initial study (days 1, 4, 7, 10, 14, 16, 19, 22, and 25). The results showed that the beneficial effects on depressive symptoms were not sustained beyond one month. Also, values of regional homogeneity assessed using functional magnet resonance imaging (fMRI) initially increased in frontal, temporal and parietal lobes after ketamine. However, this increase completely disappeared after day 79 of treatment (89). Similarly, a recent case series of six patients with SCZ reports on the effects of S-ketamine on negative symptoms (90). S-ketamine was administered subcutaneously once a week in addition to baseline antipsychotic medication, with doses ranging from 0.5 mg/kg to 1 mg/kg body weight over four weeks. The study observed a significant reduction in Brief Negative Symptom Scale (BNSS) scores (91) and a slight decrease in positive symptoms, as measured by the Brief Psychiatric Rating Scale (BPRS) (92, 93). Although limited in size, this study suggests that positive symptoms are minimally affected by ketamine administration in some individuals with SCZ (90). In summary, across the few studies investigating ketamine administration in patients with SCZ, a transient increase in symptoms such as hallucinations, delusional thinking, and thought disorder may occur. These effects typically peak within 20 minutes after infusion and resolve within 90 to 180 minutes, with no sustained symptomatology beyond three hours. Notably, in some cases, individual responses varied considerably (94–96). However, similar perceptual and cognitive short-term alterations have been observed in healthy individuals following ketamine administration, underscoring that these effects are not specific to SCZ (94).

## Conclusion

In conclusion, the reluctance to use ketamine in the treatment of patients with psychotic disorders has so far been based on concerns regarding the potential exacerbation of psychotic symptoms. However, existing evidence on the effects of ketamine in patients with psychotic conditions, although sporadic for now, does not report persistent exacerbation of positive symptoms in patients with SCZ treated with ketamine. Rather, dissociative effects occurring during ketamine administration were transient and lasted approximately 40 minutes. Observations from single case reports and some case series suggest that ketamine could be beneficial for treating negative and depressive symptoms in patients with SCZ. However, controlled clinical trials are needed to confirm these observations and for determining whether ketamine is a safe possibly effective treatment in patients with psychotic disorders.
